# Genotype-by-environment interactions govern fitness changes associated with adaptive mutations in two-component response systems

**DOI:** 10.3389/fgene.2024.1349507

**Published:** 2024-02-23

**Authors:** Brittany R. Sanders, Jordan E. Miller, Noor Ahmidouch, Joseph L. Graves, Misty D. Thomas

**Affiliations:** Department of Biology, North Carolina Agricultural and Technical State University, Greensboro, NC, United States

**Keywords:** gene-by-environment (GxE) interaction, two-component response systems, adaptation, fitness, *Escherichia coli*

## Abstract

**Introduction:** Two-component response systems (TCRS) are the main mechanism by which prokaryotes acclimate to changing environments. These systems are composed of a membrane bound histidine kinase (HK) that senses external signals and a response regulator (RR) that activates transcription of response genes. Despite their known role in acclimation, little is known about the role TCRS play in environmental adaptation. Several experimental evolution studies have shown the acquisition of mutations in TCRS during adaptation, therefore here we set out to characterize the adaptive mechanism resulting from these mutations and evaluate whether single nucleotide changes in one gene could induce variable genotype-by-environment (GxE) interactions.

**Methods:** To do this, we assessed fitness changes and differential gene expression for four adaptive mutations in *cusS*, the gene that encodes the HK CusS*,* acquired by *Escherichia coli* during silver adaptation.

**Results:** Fitness assays showed that as the environment changed, each mutant displayed a unique fitness profile with greatest fitness in the original selection environment. RNAseq then indicated that, in ± silver nitrate, each mutant induces a primary response that upregulates *cusS,* its RR *cusR*, and constitutively expresses the target response genes *cusCFBA*. This then induces a secondary response via differential expression of genes regulated by the CusR through TCRS crosstalk. Finally, each mutant undergoes fitness tuning through unique tertiary responses that result in gene expression patterns specific for the genotype, the environment and optimized for the original selection conditions.

**Discussion:** This three-step response shows that different mutations in a single gene leads to individualized phenotypes governed by unique GxE interactions that not only contribute to transcriptional divergence but also to phenotypic plasticity.

## Introduction

Microbes are an attractive platform for evaluating complex genetic interaction as they respond to broad and diverse environmental changes quickly and continuously in nature (Sabarly et al., 2016). To accomplish this, they either display phenotypic plasticity or undergo genotypic adaptation (Sabarly et al., 2016). Phenotypic plasticity or acclimation occurs when prokaryotes manipulate behavior, morphology or metabolism thereby promoting survival via changes in gene expression. Alternatively, they can undergo genetic adaptation due to natural selection (Moxon ER et al., 1994; Foster, 2000). Often the behavioral, morphological, or metabolic changes observed during acclimation end up becoming the targets of selection (Rodriguez-Verdugo et al., 2016).

Two-component response systems (TCRS) make up the most widely used mechanism by which prokaryotes sense, respond, and acclimate to external factors to initiate phenotypic plasticity (Hoch, 2000; Zschiedrich CP et al., 2016; Schaefers et al., 2017). They have been shown to play a role in a variety of essential cellular processes including cell division, metabolism, pathogenicity, antibiotic resistance, and motility (Jacob-Dubuisson et al., 2018). Their role in environmental acclimation may explain why free-living bacteria have evolved many more TCRS as compared to their pathogenic counterparts (Koretke KK et al., 2000).

TCRS facilitate physiological acclimation via two proteins (West and Stock, 2001), a histidine kinase (HK) which senses an external stimulus and relays that message to the interior of the cell through autophosphorylation. The HK then transfers the phosphate to a response regulator (RR), which then regulates transcription of target genes involved in the cellular response and subsequent acclimation (West and Stock, 2001; Zschiedrich CP et al., 2016). Even though TCRS are one of the best studied systems for acclimation in prokaryotes, very little is known about their role in adaptation.

TCRS play a role in prokaryotic adaptation to both ionic and nanoparticle silver. The latter have been hailed as a new method to address multidrug resistant organisms and unfortunately, the vast majority of studies employing metallic or metallic oxide against bacteria have not considered, or have completely underestimated, the potential for the evolution of resistance (Gupta and Silver, 1998; Graves et al., 2015). Silver’s mechanism of action is multiform, it interacts with the cell wall and membrane binding to thiol groups in respiratory enzymes and other proteins causing them to become inactivated and disrupting metabolism. The penetration of silver into the cell also is thought to disrupt cell signaling, DNA replication, transcription, translation, and cell division. This may occur directly or may occur via the generation of reactive oxygen species (ROS) (Liau SY et al., 1997; Rai et al., 2009). Three previous studies used experimental evolution (EE) to demonstrate rapid adaptation to both silver nanoparticles and ionic silver in *Escherichia coli* (Graves et al., 2015; Randall et al., 2015; Tajkarimi et al., 2017). Whole genome resequencing then demonstrated that mutations in the genes *cusS*, *ompR*, *rpoB*, and *purL* were important contributors to this response. The CusS/R TCRS is one of the main mechanisms by which gram-negative bacteria sense and respond to both silver and copper in the environment (Gudipaty and McEvoy, 2014). Upon activation, the HK CusS autophosphorolates, which in turn, stimulates phosphortransfer to the RR CusR. CusR then activates transcription of the *cusCFBA* operon, leading to acclimation via expression of a copper/silver efflux pump. This system allows the cell to maintain cellular homeostasis and acclimate to low levels of silver within its environment (Gudipaty and McEvoy, 2014). Mapping *cusS* mutations from all three experiments, show that they cluster in two regions; near the first transmembrane domain in the N-terminal cytoplasmic tail, and in the HAMP and ATPase domain of the cytoplasmic kinase core. This clustering may be important for adaptation, and therefore, the ability to understand the impact of these mutations on function and fitness may allow us to better predict which mutations may lead to a persistent phenotype if they are acquired in nature.

These EE studies also support the idea that several possible mutations will be acted upon by positive natural selection to confer resistance in a particular environment and it is likely that not all these variants will confer the same level of resistance. Furthermore, we expect that the fitness of these variants will differ by environment (Zar, 1999; Martin and Lenormand, 2015; Gifford et al., 2016; Soley et al., 2023). This phenomenon is known as genotype-by-environment (GxE) interaction which are the non-additive effects of an organism’s genotype and environment on the expression of a trait (Remold and Lenski, 2001). GXE is ubiquitous in nature. It has been shown that there are several different types of GxE interactions, including: where the genotypic rank order is preserved relative to the fitness outcome in different environments, and where the rank order is not maintained relative to the fitness outcome (Remold and Lenski, 2001). This may then impose varying fitness effects on each of the different mutants, in addition to varying secondary effects (pleiotropy) which can then have an impact on global regulation. Due to pleiotropy, it is difficult to predict specific GxE interactions. As a result, a better understanding of GxE remains a fundamental goal in creating a cross-bridge between ecology, genetics, and evolution (Jin and Gross, 1989; Remold and Lenski, 2001; Qi et al., 2014; Pietsch et al., 2016; Ostrer et al., 2021) and, in deepening our understanding of these interactions, this may shed light on the process of selection for adaptive phenotypes (Hall, 2013).

The overall goal of this study is to characterize the adaptive mechanism resulting from natural selection on TCRS and to demonstrate the biological consequences on both function and fitness of populations that have acquired these mutations (Bennett and Lenski, 1997; Schrag SJ et al., 1997; Kassen and Bataillon, 2006). We examined whether GxE interactions influence the fitness of individuals carrying different mutations in the same gene and if adaptive mutations in the same/different protein domains show similarities or differences in their GxE interactions. We answered these questions by evaluating four adaptive mutations, in three different domains of the HK *cusS* that have been shown to be involved in the evolution of silver resistance. These mutations were put into the wild type (WT) *E. coli* K12 MG1655 (the original selection strain) to eliminate the effects of varying genetic backgrounds that are typically acquired during selection. We then assessed fitness changes relative to the WT in two different types of media differing by nutrient content, in both broth and on agar, across varying silver nitrate concentrations deviating from the initial selection environment in which these variants arose. While EE studies have shown that there are multiple possible *cusS* mutations that confer silver resistance, it is likely that not all these mutations confer the same level of resistance, nor fitness, and these unique phenotypes may be due to varying GxE interactions.

## Methods

### Strains and growth conditions

*Escherichia coli* K-12 MG1655 (ATCC # 10798D-5) was used as our WT strain as it was the ancestral strain used in previous EE studies where our mutants were identified (Graves et al., 2015; Tajkarimi et al., 2017). All growth experiments were cultured in Luria Broth (Lennox) (Carolina Biology; Burlington, NC – Item #21-6710) and/or Davis Minimal Broth (DMB) (Difco Laboratories-BD; Sparks, MD- Ref # 275610) supplemented with 10% dextrose as a carbon source, overnight at 37°C with continuous shaking at 200 rpm unless otherwise noted.

### Insertion of *cusS* chromosomal mutations

We previously published the design and construction of these variants followed by whole genome sequencing for mutant and off-target validation (Sanders et al., 2023). In short, we designed ssDNA oligonucleotides to insert *cusS* mutations (R15L, T14P, T17P and N279H) into the chromosome of *Escherichia coli* K12 MG1655 using the Mage Oligo Design Tool (MODEST) (Bonde et al., 2014). We then followed a standard protocol for recombineering (Sawitzke et al., 2013). The only experimental deviation was in the final step, as our desired *cusS* mutations had been shown to lead to silver resistance, we performed a 10-fold serial dilution and plated on 20 μg/mL of silver nitrate to screen for mutations. We then extracted genomic DNA using the OMEGA E.Z.N.A.® Bacterial DNA Kit (Omega Bio-tek, Inc., GA, USA), PCR amplified the *cusS* gene and sent for sequencing (ETON Biosciences, Durham, NC, USA). After mutations were confirmed, the plasmid was cured by serial plating on LB alone, typically it took only 1–2 overnight platings to cure the plasmids. We then extracted genomic DNA from the cured populations and performed whole genome Illumina sequencing at the Microbial Genome Sequencing Center (MiGS Center) at the University of Pittsburgh (Sanders et al., 2023).

### Changes in fitness assayed in broth

Twenty-four-hour growth assays were conducted using *E. coli* K12-MG1655 carrying the WT sequence of *cusS* and each of the four *cusS* mutants (R15L, T14P, T17P and N279H) in the presence of increasing concentrations of silver nitrate. To begin, archived glycerol stocks of each population were inoculated in either LB media or DMB media supplemented with 10% dextrose overnight at 37°C with continuous shaking at 200 rpm. Overnight cultures were then diluted to an OD_600nm_ of 0.05 for normalization and added to a 96-well plate containing a concentration gradient of silver nitrate, 0-5,000 ng/mL in LB or (0–100 ng/mL in DMB. Plates were then covered with an optically clear MicroAmp Optical Adhesive film (ThermoFisher®) and incubated for 24 h in a Varioskan Lux 96-well plate reader at 37°C with shaking. Measurements were read at an OD_600nm_ every hour for 24 h. Each population was assessed in triplicate. Data was then normalized using the negative controls and the mean values were then subtracted from each replicate population for each concentration and plotted in Graph Pad Prism version 8.0.0 for Mac OS X (Graph Pad Software, San Diego, CA, USA). For each growth curve we then performed a linear regression on log phase growth to calculate the slope and *p*-values for differences between each mutant and the WT in the same environment. We also performed an Analysis of Variance (ANOVA) utilizing the General Linear Models in IBM SPSS v29 to evaluate changes in final 24-hour optical densities and reported *p*-values. To determine fitness changes relative to the WT, we calculated the area under the curve (AUC) for the 24-hour growth curves (Soley et al., 2023), the AUC along with the standard error and *N* (*N* is defined as *df* + 1 where *df* is the number of data points for that group minus the number of hours) were then plotted. A one-way ANOVA was then used to calculate statistical difference between each AUC to 1) the WT in DMB, 2) the WT in the same respective environment or 3) to their respective mutant in their respective media in absence of silver nitrate, we then reported *p*-values.

### Changes in fitness on agar

Survival curve assays for the WT and each mutant (R15L, T14P, T17P and N279H) were performed and scored based on previous methods (Tajkarimi et al., 2017). Briefly, all mutant populations were grown up overnight in DMB or LB, the following day these cultures were diluted 1:100 and grown to an OD_600_ of 0.8. Each culture was then serial diluted from 1 to 10^–7^ for a total of eight dilutions. 5 µL of each dilution was spotted twice onto DMB or LB agar plates supplemented with increasing concentration (0–100 ng/mL for DMB and 0-50,0000 ng/mL for LB) of silver nitrate and incubated for 24 h at 37°C. Growth was then scored on a scale from 0 to 8 based on the highest dilution at which growth was observed and converted to percent cell survival as previously described [12], all experiments were performed in triplicate. To then calculate changes in fitness relative to the WT, we used PRISM to calculate the row statistics for each mutant at each concentration of silver, this provided us with a mean, standard error, and *N*. These values were then used as our base values for calculating changes in fitness. A one-way ANOVAs was then used to evaluate changes in fitness relative to 1) the WT in DMB, 2) the WT in the same respective environment or 3) to their respective mutant in their respective media in absence of silver nitrate and *p*-values were reported.

### RNAseq

Cultures of the WT and each mutant (in LB and DMB) were grown overnight. The next day each population was subcultured 1:100 six times (for each type of media) and left to grow to an O.D. of 0.2 in DMB and 0.5 in LB. Once they reached the desired optical density, three of each population in each media were left to incubate for 1 h, then pooled, pelleted, and stored at −80°C. The additional three populations remaining were exposed to silver nitrate (DMB = 5 ng/mL and LB = 10,000 ng/mL) for 1 h, then pooled, pelleted, and stored at −80°C. Frozen pellets were then shipped to SeqCenter (seqcenter.com) for RNA extraction, RNAseq and differential analysis. At SeqCenter, samples were DNAse treated with Invitrogen DNAse (RNAse free). Library preparation was performed using Illumina’s Stranded Total RNA Prep Ligation with Ribo-Zero Plus kit and 10bp IDT for Illumina indices. Sequencing was done on a NextSeq2000 giving 2x51bp reads. Demultiplexing, quality control, and adapter trimming was performed with bcl-convert (v3.9.3) [1 – bcl-convert]. Average reads per sample exceeded 20M and % bp > Q30 were all ∼94% (Illumina. BCL Convert, 2021). Read mapping was performed with HISAT2 (Zhang et al., 2021). Read quantification was performed using Subread’s featureCounts (Liao et al., 2013) functionality. Read counts loaded into R (Ihaka and Gentleman, 1996) and were normalized using edgeR’s (Robinson et al., 2010) Trimmed Mean of M values (TMM) algorithm. Subsequent values were then converted to counts per million (cpm). Differential expression analysis was performed using edgeR’s exact test for differences between two groups of negative-binomial counts with an estimated dispersion value of .1. Finally, pathway analysis was performed using limma’s (Ritchie et al., 2015) “kegg” functionality. The genes that were considered Up/Down in this analysis were at FDR <.05.

## Results

**FIGURE 1:**
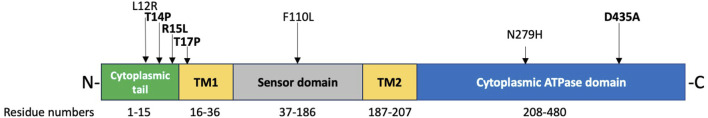
Linearized model of the CusS protein and location of adaptive mutations. The CusS monomer can be divided into 5 major subdomains. First, the N-terminal cytoplasmic tail which is composed of the first 15 residues and houses three silver adaptive mutations (L12R, T14P and R15L), two of which are represented in this study. The second domain is the first transmembrane segment (TM1); to date, only the T17P adaptive mutation has been identified in this domain in an EE study. This then leads into the periplasmic sensor domain; there has yet to be adaptive mutations identified in this domain due to EE studies in silver nitrate, although the Long-Term Evolution Experiment (LTEE) did identify one adaptive mutation in this domain (F110L (Barrick and Lenski, 2013; Lenski, 2018)). Next is the second transmembrane domain, which to our knowledge has never acquired any adaptive mutations. Finally, we have the C-terminal kinase domains (which also houses a HAMP domain and a dimerization domain). There have been two adaptive mutations identified in this domain (D435A in the dimerization domain and N279H in the ATP binding pocket) although only N279H is represented in this study. We will note that, we attempted to generate all the mutants above, albeit were only successful at the ones in bold (T14P, R15L, T17P and N279H).

**FIGURE 2:**
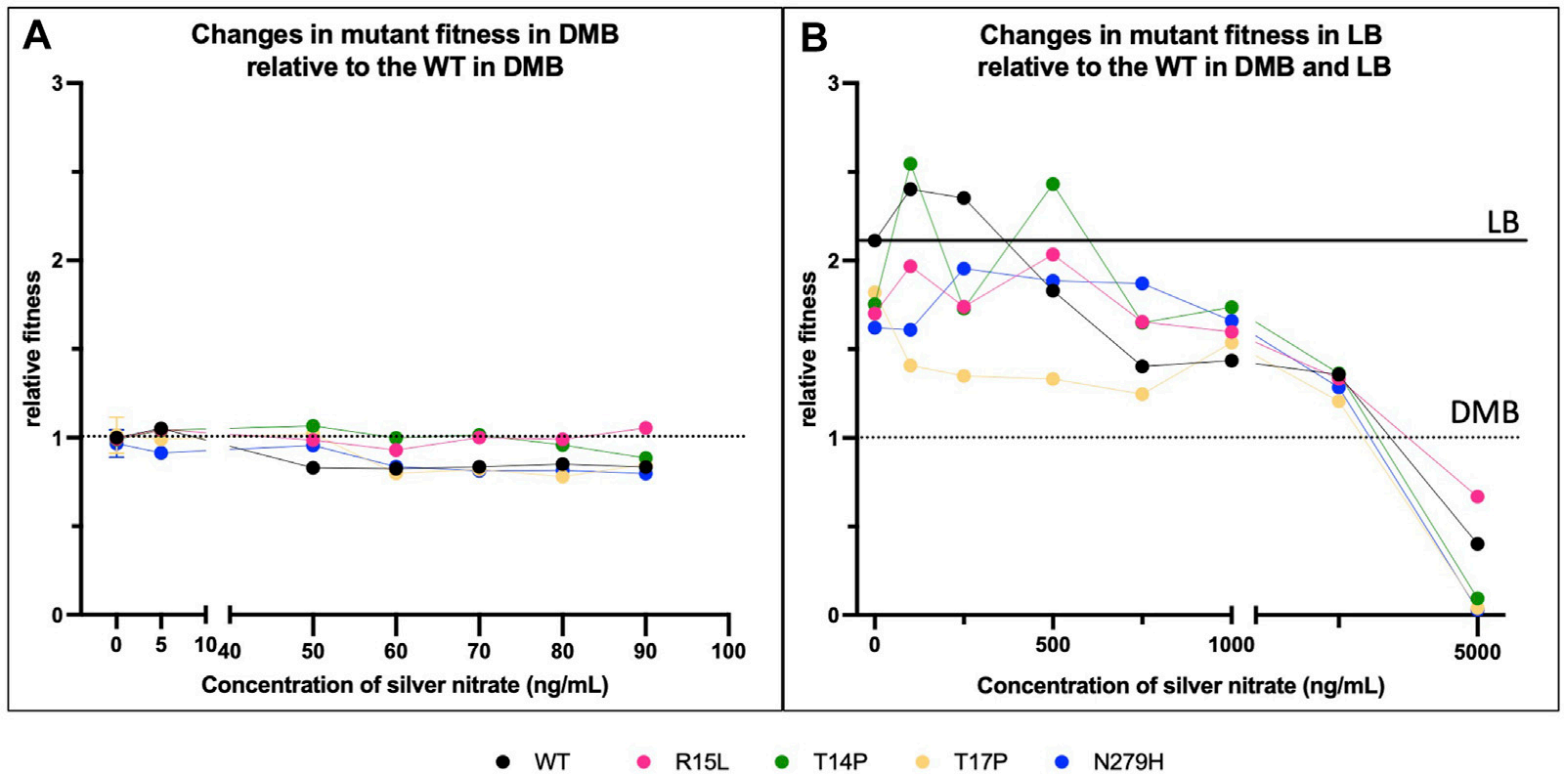
Single amino acid changes in one gene can affect organismal fitness which is further influenced by media type and silver nitrate concentrations. Changes in fitness relative to the WT in all four *cusS* mutants was evaluated in **(A)** DMB (the original selective media), in varying concentrations of silver nitrate, including the original selection concentration of 50 ng/mL, and in **(B)** LB at varying concentrations of silver nitrate. The black dotted line represents the fitness of the WT in DMB alone and the black solid line represents the fitness of the WT in LB alone. Here we show that the composition of the media has a clear effect on fitness, and interestingly, mutants show no change in fitness relative to the WT in DMB alone while all show reduced fitness as compared to the WT in LB. In addition, the overall tolerance for the selective agent, silver nitrate, is significantly higher in LB than it is in DMB. Together this data shows that mutant displays a unique fitness pattern and that is the result of each individual genotype interacting to varying degrees with the silver nitrate in its environment. *p*-values from one-way ANOVAs are given in Table 2.

In total, seven adaptive mutations have been identified in the *cusS* gene, three in the N-terminal cytoplasmic tail, one in transmembrane 1, one in the periplasmic sensor domain and two in the cytoplasmic ATPase domain (Figure 1) (Graves et al., 2015; Randall et al., 2015; Affandi et al., 2016; Tajkarimi et al., 2017; Lenski, 2018). We were successful at generating four of the seven mutants across three of these domains. We then evaluated changes in fitness to characterize phenotypic variability across environments and RNAseq to evaluate the genetic basis for these phenotypic changes. To do this, we analyzed 24-hour growth curves of our WT and four single mutants (R15L, T14P, T17P and N279H) in 30 different environments. This included two different types of liquid media (LB and DMB), across increasing concentrations of silver nitrate (0–100 ng/mL in DMB and 0–5,000 ng/mL in LB) for a total of 16 environments. We then evaluated survival on two types of solid agar (DMB and LB) again with increasing concentrations of silver nitrate (0–100 ng/mL in DMB and 0–50,000 ng/mL in LB) for an additional 14 environments. The original selection environment for three of the mutants (R15L, T14P and N279H) was DMB broth supplemented with 50 ng/mL silver nitrate (Tajkarimi et al., 2017) which was included as one of our assayed environments. One of the mutants (T17P) was selected in Mueller Hinton broth supplemented with 1,000 ng/mL silver nitrate (Randall et al., 2015) which was not included as one of our growth environments. 24-hours was chosen for growth assays as this was the endpoint for subculturing in the original EE studies (Tajkarimi et al., 2017) that led to selection of these *cusS* mutations. All 24-hour growth curves can be found in the Supplementary Figures S1, S2. To determine changes in fitness, we calculated the area under the curve (AUC) for each growth curve. We used AUC as a proxy for fitness as it represents all the features of the bacterial growth curve, including lag time, the slope of exponential phase (growth rate) and the final optical density (24-hour cell yield) as a single comparable metric (Gifford et al., 2016; Dunai et al., 2019; Shea et al., 2020; Arrieta-Ortiz et al., 2023; Soley et al., 2023). We also calculated each of the three individual metrics and they are reported in Table 1. In DMB, the AUC of each population, at each concentration of silver nitrate, was divided by the AUC for the WT in DMB alone (Figure 2A, black dotted line). For LB we divided the AUC of each population by the WT in DMB alone (Figure 2B, black dotted line) and by that of WT in LB alone (Figure 2B, black solid line). One-way ANOVAs were calculated to validate changes in fitness relative to the WT (Table 2A - DMB vs. DMB; Table 2D - DMB vs. LB and Table 2E - LB vs. LB). One-way ANOVAs were also used to determine if the changes in fitness were the result of the specific genotype (Table 2B - DMB; Table 2F - LB) by comparing each mutant to the WT in the same respective silver concentration, or due to the presence of silver nitrate (Table 2C - DMB and Table 2G - LB) by comparing each population to itself in absence of silver nitrate.

**TABLE 1:** 24-hour growth curve metrics and statistics in both DMB and LB broth in increasing concentrations of silver nitrate.

DMB						LB					
	Lag time	Log phase slope	*p*-Value	24-hour O.D.600	*p*-Value		Lag time	Log phase slope	*p*-Value	24-h O.D.600	*p*-Value
		Growth rate		Cell yield				Growth rate		Cell yield	
WT	2	0.051		0.334		WT	1	0.109		0.636	
R15L	2	0.047	0.216	0.356	1.000	R15L	1	0.102	0.498	0.481	<.001
T14P	2	0.064	0.003	0.335	1.000	T14P	0	0.085	0.067	0.479	<.001
T17P	2	0.060	0.032	0.309	1.000	T17P	1	0.108	0.959	0.557	0.011
N279H	2	0.059	0.058	0.279	0.217	N279H	1	0.104	0.589	0.455	<.001
5 ng/uL						100 ng/uL					
WT	2	0.075		0.319		WT	1	0.112		0.748	
R15L	2	0.064	0.026	0.338	1.000	R15L	1	0.111	0.944	0.606	<.001
T14P	2	0.058	0.002	0.344	1.000	T14P	1	0.096	0.262	0.759	1.000
T17P	2	0.075	0.904	0.324	<.001	T17P	1	0.101	0.148	0.386	<.001
N279H	2	0.065	0.006	0.310	<.001	N279H	1	0.096	0.046	0.457	<.001
50 ng/uL						250 ng/uL					
WT	5	0.043		0.273		WT	1	0.113		0.728	
R15L	3	0.054	0.115	0.324	<.001	R15L	1	0.105	0.360	0.485	<.001
T14P	2	0.069	0.004	0.369	<.001	T14P	0	0.084	0.022	0.468	<.001
T17P	2	0.068	0.006	0.259	<.001	T17P	1	0.098	0.061	0.372	<.001
N279H	2	0.065	0.010	0.284	<.001	N279H	1	0.115	0.778	0.589	<.001
60 ng/uL						500 ng/uL					
WT	2	0.031		0.323		WT	1	0.114		0.574	
R15L	2	0.033	0.550	0.352	<.001	R15L	1	0.120	0.519	0.610	0.598
T14P	2	0.028	0.556	0.369	<.001	T14P	0	0.079	0.004	0.759	<.001
T17P	2	0.027	0.189	0.275	0.071	T17P	2	0.102	0.092	0.378	<.001
N279H	2	0.033	0.404	0.268	0.866	N279H	1	0.125	0.123	0.578	1.000
70 ng/uL						750 ng/uL					
WT	2	0.031		0.284		WT	2	0.086		0.407	
R15L	2	0.036	0.829	0.340	1.000	R15L	1	0.085	0.982	0.465	<.001
T14P	2	0.033	0.935	0.367	1.000	T14P	1	0.088	0.906	0.468	<.001
T17P	2	0.028	0.889	0.256	<.001	T17P	3	0.114	0.070	0.364	0.014
N279H	2	0.031	0.991	0.270	<.001	N279H	2	0.114	0.094	0.592	<.001
80 ng/uL						1,000 ng/uL					
WT	2	0.034		0.283		WT	1	0.111		0.566	
R15L	2	0.040	0.088	0.372	<.001	R15L	2	0.098	0.111	0.459	<.001
T14P	2	0.032	0.604	0.312	<.001	T14P	1	0.111	0.932	0.473	0.535
T17P	2	0.027	0.016	0.288	<.001	T17P	1	0.096	0.073	0.420	0.001
N279H	2	0.032	0.542	0.253	0.319	N279H	1	0.112	0.907	0.452	0.009
90 ng/uL						2,500 ng/uL					
WT	2	0.028		0.283		WT	5	0.097		0.532	
R15L	2	0.037	0.047	0.372	<.001	R15L	5	0.104	0.589	0.444	1.000
T14P	2	0.031	0.437	0.312	0.002	T14P	4	0.083	0.265	0.446	1.000
T17P	2	0.026	0.604	0.288	1.000	T17P	4	0.078	0.188	0.389	0.002
N279H	2	0.030	0.716	0.253	0.166	N279H	5	0.111	0.309	0.436	0.627
						5,000 ng/uL					
						WT	12	0.061		0.246	
						R15L	8	0.065	0.934	0.354	<.001
						T14P	18	0.015	0.131	0.082	1.000
						T17P	N/A	N/A	N/A	N/A	N/A
						N279H	N/A	N/A	N/A	N/A	N/A

**TABLE 2:** Calculated p-values resulting from one-way ANOVAS to validate changes in fitness relative to the WT in broth (Table 2A - DMB vs DMB and Table 2D - DMB vs. LB and Table 2E - LB vs LB). One-way ANOVAs were also used to determine if the changes in fitness were the result of the specific genotype (Table 2B - DMB and Table 2F - LB) by comparing each mutant to the WT in the same respective silver concentration, or due to the presence of silver nitrate (Table 2C - DMB and Table 2G - LB) by comparing each population to itself in absence of silver nitrate.

A	vs. WT in DMB [0]					B	vs. [WT] in DMB					C	vs. mutant @ [0] in DMB				
DMB						DMB						DMB					
[silver nitrate] ng/mL	WT	R15L	T14P	T17P	N279H	[silver nitrate] ng/mL	WT	R15L	T14P	T17P	N279H	[silver nitrate] ng/mL	WT	R15L	T14P	T17P	N279H
0	**>0.9999**	0.9899	0.9312	0.828	0.1167	0	**>0.9999**	0.9813	0.9092	1	0.1314	0	**>0.9999**	**>0.9999**	**>0.9999**	**>0.9999**	**>0.9999**
5	0.0033	0.0137	0.0331	0.9511	<0.0001	5	**>0.9999**	0.9823	0.8932	0.0006	<0.0001	5	0.0298	0.0001	0.0843	0.6449	0.0011
50	<0.0001	0.9449	0.0029	0.6868	0.1006	50	**>0.9999**	0.0001	0.0001	0.0001	0.0001	50	<0.0001	0.9964	0.0025	0.9986	0.9679
60	<0.0001	<0.0001	>0.9999	<0.0001	<0.0001	60	**>0.9999**	<0.0001	<0.0001	0.0668	0.7069	60	<0.0001	<0.0001	0.998	<0.0001	<0.0001
70	0.9998	>0.9999	0.9399	<0.0001	<0.0001	70	**>0.9999**	0.998	0.8392	<0.0001	<0.0001	70	>0.9999	0.9777	0.7044	<0.0001	<0.0001
80	<0.0001	0.9781	0.081	<0.0001	<0.0001	80	**>0.9999**	<0.0001	<0.0001	0.0006	0.1805	80	<0.0001	>0.9999	0.5936	<0.0001	<0.0001
90	<0.0001	0.0038	<0.0001	<0.0001	<0.0001	90	**>0.9999**	<0.0001	0.0129	0.8303	0.1273	90	<0.0001	<0.0001	<0.0001	<0.0001	<0.0001
100	<0.0001	<0.0001	<0.0001	<0.0001	<0.0001	100	**>0.9999**	0.0496	0.7324	0.0318	<0.0001	100	<0.0001	<0.0001	<0.0001	<0.0001	<0.0001

D	vs. WT in DMB [0]					E	vs. WT in LB [0]					F	vs. [WT] in LB					G	vs. mutant @ [0] in LB				
LBvsDMB						LBvsLB						LBvsLB						LBvsLB					
[silver nitrate] ng/mL	WT	R15L	T14P	T17P	N279H	[silver nitrate] ng/mL	WT	R15L	T14P	T17P	N279H	[silver nitrate] ng/mL	WT	R15L	T14P	T17P	N279H	[silver nitrate] ng/mL	WT	R15L	T14P	T17P	N279H
0	<0.0001	<0.0001	<0.0001	<0.0001	<0.0001	0	**>0.9999**	0.0007	0.0041	0.0267	<0.0001	0	**>0.9999**	0.0007	0.0041	0.0267	<0.0001	0	**>0.9999**	**>0.9999**	**>0.9999**	**>0.9999**	**>0.9999**
100	<0.0001	<0.0001	<0.0001	0.001	<0.0001	100	0.1012	0.686	0.0044	0.0001	0.0006	100	**>0.9999**	0.0011	0.5699	<0.0001	<0.0001	100	0.1538	0.0007	<0.0001	<0.0001	>0.9999
250	<0.0001	<0.0001	<0.0001	0.0004	<0.0001	250	0.1265	0.0043	0.0033	0.0001	0.4744	250	**>0.9999**	<0.0001	<0.0001	<0.0001	0.0001	250	0.3331	0.9957	>0.9999	<0.0001	<0.0001
500	<0.0001	<0.0001	<0.0001	0.0259	<0.0001	500	0.1595	0.9717	0.0907	0.0001	0.3364	500	**>0.9999**	0.3363	<0.0001	0.0007	0.9817	500	0.1732	<0.0001	<0.0001	<0.0001	0.0026
750	<0.0001	<0.0001	<0.0001	<0.0001	<0.0001	750	0.0001	0.0001	0.0001	0.0001	0.0291	750	**>0.9999**	0.0001	0.0002	0.0301	<0.0001	750	<0.0001	0.9822	0.6858	<0.0001	0.0053
1,000	<0.0001	<0.0001	<0.0001	<0.0001	<0.0001	1,000	0.0143	0.0001	0.0003	0.0001	0.0001	1,000	**>0.9999**	0.0015	0.371	<0.0001	0.0283	1,000	0.191	0.511	>0.9999	<0.0001	0.9977
2,500	<0.0001	<0.0001	<0.0001	<0.0001	<0.0001	2,500	0.0001	0.0001	0.0001	0.0001	0.0001	2,500	**>0.9999**	0.9746	0.9997	0.0132	0.427	2,500	<0.0001	<0.0001	<0.0001	<0.0001	<0.0001
5,000	<0.0001	<0.0001	<0.0001	<0.0001	<0.0001	5,000	0.0001	0.0001	0.0001	0.0001	0.0001	5,000	**>0.9999**	0.0031	0.0005	<0.0001	<0.0001	5,000	<0.0001	<0.0001	<0.0001	<0.0001	<0.0001
10,000	<0.0001	<0.0001	<0.0001	<0.0001	<0.0001	10,000	0.0001	0.0001	0.0001	0.0001	0.0001	10,000	**>0.9999**	>0.9999	<0.0001	<0.0001	<0.0001	10,000	<0.0001	<0.0001	<0.0001	<0.0001	<0.0001

The population used for comparison in the one-ways ANOVAs for the mutants and conditions.

### Individual mutants display variable fitness across silver nitrate concentrations with optimal fitness in the original selective environment

To begin, we evaluated if changes in fitness were associated with 1) changes in genotype and 2) the environment via increasing concentrations of silver nitrate in DMB (Figure 2A). DMB +50 ng/mL was the original selection medium for R15L, T14P and N279H. When comparing the WT and each of the mutants in DMB alone, they all show no change in fitness albeit two mutants (T14P and T17P) do show a slight increase in growth rate over the WT but not enough to significantly change the AUC (Table 1). With the addition of 5 ng/mL silver nitrate, WT, R15L and T14P all show a slight increase in fitness relative to the WT in DMB, which appears to be influenced by the presence of silver nitrate itself (Table 2C). When comparing the mutants to WT at this same concentration, T17P maintains starting fitness due to an increase in final O.D., while N279H shows a reduction in fitness associated with a decrease in both growth rate and final O.D. At 50 ng/mL, the fitness of the WT decreases while T14P increases and R15L, T17P and N279H maintain similar fitness to the WT in DMB alone. This fitness advantage is the result of higher 24-hour O.D. values for R15L and both a higher ODs and growth rate for T14P, T17P and N279H. Once above 50 ng/mL, the fitness of the WT decreases, until 100 ng/mL where it can no longer survive. T17P and N279H show reduced fitness below that of the WT at the same concentrations until again 100 ng/mL where they no longer grow. R15L and T14P retain their starting fitness until 90 ng/mL by maintaining higher final O.D., where T14Ps fitness is finally reduced by silver nitrate. As with the others, the growth of these two is completely inhibited at 100 ng/mL. Together this data shows that each of these individual mutations lead to variable fitness patterns that display changing fitness ranks (Supplementary Figure S3) across increasing concentrations of silver nitrate. These fitness changes are dictated by both changes in growth rate and in 24-hour O.D. values which also vary across mutants and silver nitrate concentrations. Finally, the original selection condition is the only environment in which all mutants show increased fitness over the WT.

### Individual mutants display variable fitness across different media types

To characterize the fitness effects associated with changing from DMB, a minimal media and the original selection media, to that of a new rich media, LB, we compared fitness for each of the populations at each concentration of silver nitrate to that of the WT in DMB alone (Figure 2B, black dotted line; Table 2D). When the WT and all mutants are grown in LB and LB + silver nitrate, there is a general increase in fitness across all concentrations, until we reach 5,000 ng/mL where the concentration is high enough to reduce the fitness of all populations relative to the WT until 10,000 ng/mL where they all ceased to grow. We then continued by comparing fitness of each mutant to that of the WT in LB alone (Figure 2B, solid black line; Table 2E) to determine if the concentration and genotypic effect in LB were similar to what was observed in DMB. In LB, WT shows no change in fitness until 750 ng/mL where the concentration of silver nitrate is high enough to continually reduce fitness, until growth is inhibited at 10,000 ng/mL. In LB alone, all mutants show a significant decrease in fitness as compared to the WT and this is marked by a reduction in 24-hour O.D. values. At low concentrations of silver nitrate (100–500 ng/mL), R15L increases in fitness to that of the WT in LB alone, until it reaches 750 ng/mL where silver nitrate reduces fitness. Albeit it remains above that of the WT at the same concentrations by maintaining higher final O.D. values until 2,500 ng/mL where the concentration of silver nitrate becomes the primary influencer, reducing fitness until cells no longer grow at 10,000 ng/mL. At 100 ng/mL, T14P shows an increase in fitness, then decreases in fitness at concentrations above 500 ng/mL, albeit again, staying above that of the WT in the same concentration due to an increased final O.D. Above 1,000 ng/mL fitness continues to reduce until it can no longer survive at 10,000 ng/mL. T17P (which was selected for in a rich media, MHB) shows reduced fitness not only below that of the WT in LB alone, but also below that of the WT at each respective concentration. This is until we reach 1,000 ng/mL (the original selection concentration for T17P) where its fitness rises above that of the WT. Finally at 2,500 ng/mL and above, T17P again shows reduced fitness below that of the WT with lower O.D. values. N279H has reduced fitness at both 0 and 100 ng/mL from 250–500 ng/mL its fitness increases to the level of the WT in LB alone. At 750–1,000 ng/mL, silver does slightly reduce its fitness, although it remains above the WT at this same concentration. Above this, the concentration fitness is reduced until again, it can no longer grow at 10,000 ng/mL. As anticipated, this shows that the composition of the media has a clear effect on fitness that is primarily affected by changes in final O.D. Mutants show no change in fitness relative to the WT in DMB alone while all show reduced fitness as compared to the WT in LB. The overall tolerance for the selective agent, silver nitrate, is higher in LB (0–5,000 ng/mL) than it is in DMB (0–90 ng/mL), and the fitness of each specific genotype is influenced by each concentration of silver nitrate in a different way. The consistent trend across the data continues to show that as we change the genotype and deviate from the original selection environment, whether that be the specific concentration of silver nitrate and/or the media composition, changes in fitness vary between mutants and there is no maintenance of fitness rank (Supplementary Figure S3). As with the DMB selected mutants, here the one mutant (T17P) that was selected for in right media showed increased fitness above the WT only at its original selection concentration (1,000 ng/mL).

### Individual mutants display variable fitness across different media states

**FIGURE 3:**
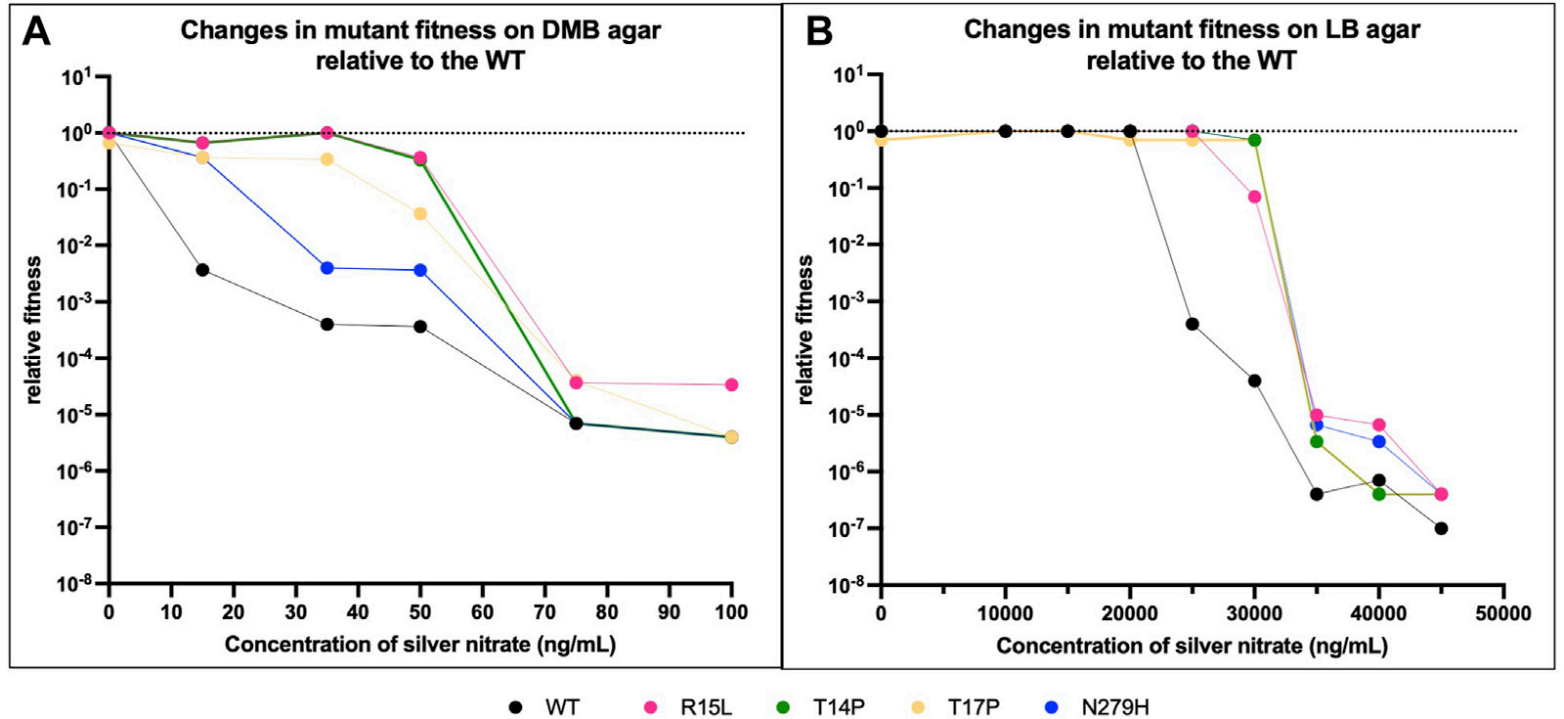
Media state influences fitness. We then evaluated if the state of the media (solid vs. liquid) had an influence on fitness relative to the WT. **(A)** shows changes in fitness on DMB agar and **(B)** on LB agar, again both with increasing concentrations of silver nitrate. The black dotted line represents the fitness of the WT in DMB in absence of silver nitrate. The black solid line represents the fitness of the WT in LB in presence of silver nitrate. Overall, the data shows that on agar as with broth, the individual genotypes interact to varying extents on the solid media (DMB vs. LB agar) and the varying concentration of silver nitrate. *p*-values from one-way ANOVAs are given in Table 3.

Next, we evaluated the changes in fitness of our populations after changing media state, using DMB and LB agar instead of broth (Figure 3; Table 3). To do this, we performed survival assays based on previously reported protocols (Affandi et al., 2016; Tajkarimi et al., 2017). After performing serial dilutions on plates, growth was scored and assigned a corresponding survival percentage, changes in fitness were then calculated by dividing survival percentages of each population by the survival percentage of the WT on each respective agar in absence of silver. For LB we did not directly compare survival percentages back to the WT on DMB agar (as we did in broth) as the survival percentage for both media types in absence of silver nitrate is 100%. One-way ANOVAs were then used to determine if there was a change in fitness from the WT in absence of silver nitrate on the respective media types (Tables 3A, D), and to determine if this difference was the result of the genotype (Tables 3B, E) or due to the silver nitrate (Tables 3C, F).

**TABLE 3:** Calculated p-values resulting from one-way ANOVAs to determine change in fitness from the WT in absence of silver nitrate on the respective agar media types (Table 3A, D), and to determine if this difference was the result of the genotype (Table 3B, E) or due to the silver nitrate (Table 3C, F).

A	vs. WT in DMB [0]					B	vs [WT] in DMB					C	vs mutant @ [0] in DMB				
DMB						DMB						DMB					
[silver nitrate] ng/mL	WT	R15L	T14P	T17P	N279H	[silver nitrate] ng/mL	WT	R15L	T14P	T17P	N279H	[silver nitrate] ng/mL	WT	R15L	T14P	T17P	N279H
0	**>0.9999**	0.9899	0.9312	0.828	0.1167	0	**>0.9999**	0.9813	0.9092	1	0.1314	0	**>0.9999**	**>0.9999**	**>0.9999**	**>0.9999**	**>0.9999**
5	0.0033	0.0137	0.0331	0.9511	<0.0001	5	**>0.9999**	0.9823	0.8932	0.0006	<0.0001	5	0.0298	0.0001	0.0843	0.6449	0.0011
50	<0.0001	0.9449	0.0029	0.6868	0.1006	50	**>0.9999**	0.0001	0.0001	0.0001	0.0001	50	<0.0001	0.9964	0.0025	0.9986	0.9679
60	<0.0001	<0.0001	>0.9999	<0.0001	<0.0001	60	**>0.9999**	<0.0001	<0.0001	0.0668	0.7069	60	<0.0001	<0.0001	0.998	<0.0001	<0.0001
70	0.9998	>0.9999	0.9399	<0.0001	<0.0001	70	**>0.9999**	0.998	0.8392	<0.0001	<0.0001	70	>0.9999	0.9777	0.7044	<0.0001	<0.0001
80	<0.0001	0.9781	0.081	<0.0001	<0.0001	80	**>0.9999**	<0.0001	<0.0001	0.0006	0.1805	80	<0.0001	>0.9999	0.5936	<0.0001	<0.0001
90	<0.0001	0.0038	<0.0001	<0.0001	<0.0001	90	**>0.9999**	<0.0001	0.0129	0.8303	0.1273	90	<0.0001	<0.0001	<0.0001	<0.0001	<0.0001
100	<0.0001	<0.0001	<0.0001	<0.0001	<0.0001	100	**>0.9999**	0.0496	0.7324	0.0318	<0.0001	100	<0.0001	<0.0001	<0.0001	<0.0001	<0.0001

D	vs. WT in DMB [0]					E	vs WT in LB [0]					F	vs [WT] in LB					G	vs mutant @ [0] in LB				
LBvsDMB						LBvsLB						LBvsLB						LBvsLB					
[silver nitrate] ng/mL	WT	R15L	T14P	T17P	N279H	[silver nitrate] ng/mL	WT	R15L	T14P	T17P	N279H	[silver nitrate] ng/mL	WT	R15L	T14P	T17P	N279H	[silver nitrate] ng/mL	WT	R15L	T14P	T17P	N279H
0	<0.0001	<0.0001	<0.0001	<0.0001	<0.0001	0	**>0.9999**	0.0007	0.0041	0.0267	<0.0001	0	**>0.9999**	0.0007	0.0041	0.0267	<0.0001	0	**>0.9999**	**>0.9999**	**>0.9999**	**>0.9999**	**>0.9999**
100	<0.0001	<0.0001	<0.0001	0.001	<0.0001	100	0.1012	0.686	0.0044	0.0001	0.0006	100	**>0.9999**	0.0011	0.5699	<0.0001	<0.0001	100	0.1538	0.0007	<0.0001	<0.0001	>0.9999
250	<0.0001	<0.0001	<0.0001	0.0004	<0.0001	250	0.1265	0.0043	0.0033	0.0001	0.4744	250	**>0.9999**	<0.0001	<0.0001	<0.0001	0.0001	250	0.3331	0.9957	>0.9999	<0.0001	<0.0001
500	<0.0001	<0.0001	<0.0001	0.0259	<0.0001	500	0.1595	0.9717	0.0907	0.0001	0.3364	500	**>0.9999**	0.3363	<0.0001	0.0007	0.9817	500	0.1732	<0.0001	<0.0001	<0.0001	0.0026
750	<0.0001	<0.0001	<0.0001	<0.0001	<0.0001	750	0.0001	0.0001	0.0001	0.0001	0.0291	750	**>0.9999**	0.0001	0.0002	0.0301	<0.0001	750	<0.0001	0.9822	0.6858	<0.0001	0.0053
1000	<0.0001	<0.0001	<0.0001	<0.0001	<0.0001	1000	0.0143	0.0001	0.0003	0.0001	0.0001	1000	**>0.9999**	0.0015	0.371	<0.0001	0.0283	1000	0.191	0.511	>0.9999	<0.0001	0.9977
2500	<0.0001	<0.0001	<0.0001	<0.0001	<0.0001	2500	0.0001	0.0001	0.0001	0.0001	0.0001	2500	**>0.9999**	0.9746	0.9997	0.0132	0.427	2500	<0.0001	<0.0001	<0.0001	<0.0001	<0.0001
5000	<0.0001	<0.0001	<0.0001	<0.0001	<0.0001	5000	0.0001	0.0001	0.0001	0.0001	0.0001	5000	**>0.9999**	0.0031	0.0005	<0.0001	<0.0001	5000	<0.0001	<0.0001	<0.0001	<0.0001	<0.0001
10000	<0.0001	<0.0001	<0.0001	<0.0001	<0.0001	10000	0.0001	0.0001	0.0001	0.0001	0.0001	10000	**>0.9999**	>0.9999	<0.0001	<0.0001	<0.0001	10000	<0.0001	<0.0001	<0.0001	<0.0001	<0.0001

On DMB agar, the WT immediately shows a steep drop off in fitness at 10 ng/mL, this continues to drop until growth is inhibited above 100 ng/mL (Figure 3A). Three of the mutants (R15L, T14P and N279H) show no change in fitness as compared to the WT at 0, while T17P shows a slight decrease. With the addition of silver nitrate, R15L and T14P maintain WT fitness at 15-50 and 15–35 ng/mL (Table 3A, dotted black line). Above these concentrations, both mutants show a reduction in fitness until growth is inhibited above 100 ng/mL. Similar to the WT, T17P and N279H show a decrease in fitness across all concentrations of silver nitrate tested. At 35 ng/mL and below, T17P’s maintains fitness above that of the WT at the same concentrations until 50 ng/mL where fitness is reduced to the same levels as the WT at the same concentrations. Statistically, N279H shows no fitness gain over the WT at any of the concentrations evaluated (Table 3B).

On LB agar, there is no change in fitness between the WT and each mutant for all concentrations of silver nitrate below 15,000 ng/mL. The WT continues to maintain this fitness at 20,000 ng/mL and at 25,000 ng/mL, the WT begins a sharp decrease due to the presence of silver nitrate until it can no longer survive at 50,000 ng/mL. The mutants maintain WT (at 0) fitness at higher concentrations. R15L’s fitness does not decrease until 30,000 ng/mL and at 35,000 ng/mL its fitness remains above that of the WT at the same concentration; above this, silver effectively reduces growth to WT levels. T14P, T17P and N279H are not influenced by silver until 35,000 ng/mL. Overall, our data shows that on agar as with broth, the individual genotypes display varying fitness and again, rank order is not maintained across concentrations of silver nitrate (Supplementary Figure S3). Of note, the mutants are no longer able to hold their selective advantage over the WT near selection concentrations as the media state is changed (Table 3B). Indicating that fitness is not only unique for media composition and silver nitrate concentration but also media state.

### Adaptive mutations in the *cusS* HK led to upregulation in the TCRS operon and constitutive expression of the *cusCFBA* response genes.

The CusS/R TCRS activates expression of the CusCFBA efflux pump in response to silver (or copper) in the environment [12]. Despite multiple studies showing that *cusS* acquires mutations as a result of the evolution of silver resistance (both ionic and nanoparticles) in the lab [1,3,4], there has yet to be data demonstrating the biological changes that take place as a result of these adaptive mutations. To gain insight into the adaptive mechanism governing these changes, we performed RNAseq on the WT and each of our mutants in presence and absence of silver nitrate (50 ng/mL) in both DMB and LB to evaluate differential expression of the *cusS/R* TCRS and the *cusCFBA* efflux pump genes, and/or any potential changes in global regulation that may accompany changes in the target system. All log fold expression data is calculated relative to the WT in absence of silver nitrate in the respective media types.

**FIGURE 4:**
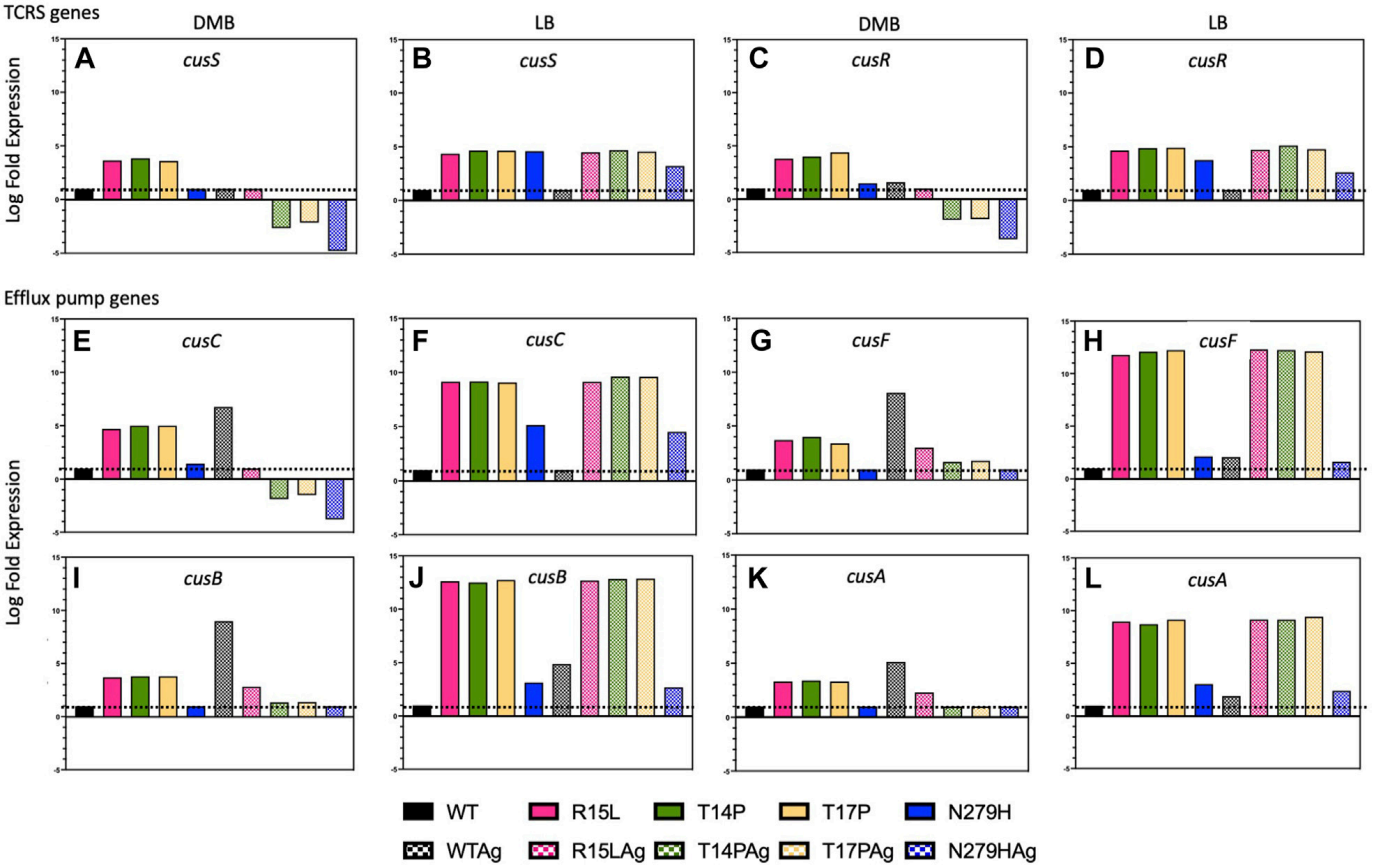
Individual *cusS* mutations lead to varying levels of both TCRS and efflux pump expression in absence of silver nitrate. Here we used RNAseq to evaluate changes in expression of both the *cusS/R* TCRS and the *cusCFBA* efflux pump genes. **(A–D)** Shows TCRS gene expression in DMB and LB in absence and presence of silver nitrate. **(E–L)** Shows expression of the individual efflux pump genes in DMB and LB, again in absence and presence of silver nitrate. The data shows that single point mutations in the *cusS* gene result in increased expression of the TCRS and constitutive expression of the efflux pump genes. In addition, the patterns of expression for these genes differ with both the individual genotype and the media type used.

In the WT, *cusR* shows a slight increase in expression in both DMB and LB with the addition of silver nitrate, while *cusS* shows no change (Figures 4A–D). In contrast, R15L, T14P and T17P all, in the absence of silver nitrate, show in DMB, a ∼3.5-fold increase and in LB a ∼4.5-fold increase over WT in expression of both genes. However, N279H in DMB showed similar levels of expression to the WT and only a ∼3.5-fold increase in expression in LB. In the presence of silver nitrate, R15L shows levels similar to WT in DMB alone while T14P, T17P and N279H all displayed decreased expression (these are not 0 but are 3-4-fold less than the WT in DMB alone). This decrease in expression of the TCRS and response genes could be due to a downregulation to meet the expression requirements and maintain fitness, alternatively, these single mutations may not be adequate to support this concentration of silver nitrate as in the original EE study the adaptive mutants held several mutations and therefore, we are observing a decrease due to cell death. In LB, R15L, T14P and T17P all show a similar ∼4.5-fold increase and N279H a ∼3-fold increase in presence of silver nitrate, similar to what was observed in its absence. Together this shows that adaptive mutations in *cusS* generally lead to upregulation in the *cusS/R* TCRS operon and this increase varies between mutants.

As expected, we also observed a significant increase in expression of all four efflux pump genes (*cusCFBA*) for the WT in DMB with the addition of silver nitrate. We also saw similar results in LB, except for *cusC* which remained at WT levels. This data confirms that this efflux pump is indeed naturally upregulated as a result of the presence of silver in the environment in both DMB and LB [13,14,34]. On the other hand, three of our mutants (R15L, T14P and T17P), in both DMB and LB, display high levels (∼3-5-fold and ∼8–12-fold respectively) of expression of all four efflux genes in absence of silver nitrate. N279H, in DMB shows no increase in efflux pump genes as compared to the WT, although in LB, show an ∼8–11-fold increase in expression over the WT in absence of silver nitrate. With the addition of silver nitrate into DMB, R15L shows only a slight increase in expression over the WT for *cusF/B/A*, whereas the other three mutants show a decrease in *cusC* and levels similar to the WT for *cusF/B/A*. In LB R15L, T14P and T17P all show similar levels of expression with silver as they do in its absence. Together this shows that single adaptive mutations in the *cusS* gene result in increased constitutive expression of the *cusCFBA* efflux pump response genes in absence of silver nitrate. In addition, the level of efflux pump expression appears to be correlated with the overexpression levels of the TCRS genes and again, varies for each mutant.

### Adaptive mutations in *cusS* partially mimic the cellular response to silver

We then investigated how these single mutations influenced expression of other biological pathways. First, we used Venny 2.1 (Oliveros, 2015) to compare differentially expressed genes in DMB vs. LB between 1) the same mutant and 2) the different mutants (Supplementary Figure S4 and Supplementary Table S1). When comparing the same mutant across DMB and LB, they only express ∼5–10% of genes in common. Of these common overlapping genes, only 2 genes (*cusR* and *cusC*) were in common among all mutants across both types of media. If we then compare all of the mutants in DMB alone, we find 6 overlapping genes (*wcaL, copA, cueO, ghxP, cusR* and *cusC*) and if we only look at the three N-terminal mutants, they show an additional 8 genes of overlap (*hprR/S, pheP, hiuH, cusS* and *cusF/B/A*). In LB, there are 23 overlapping genes (*zraP, yaaX, ugpB, yfjY, astE/B, pspG, hycA/D/B, ymcF/E, cspG, hprR, pheP, cusS/R/C/F/B/A, hiuH* and *yjcS*). Showing that in each respective media there is a common response across the mutants as they differentially express several genes in common, although, the majority of the differentially expressed genes are not only unique to each mutant but also unique for each environment.

In the presence of silver nitrate, the WT only has ∼7% of genes overlapping between DMB and LB (Supplementary Table S1). These numbers are more variable for each mutant, with R15L showing ∼5%, T14P ∼10%, T17P 20% and N279H ∼40% overlap between DMB and LB. If we then compare the shared response between the WT and all of the mutants in DMB and LB we find 20 genes in common (*pdhR, cueO, copA, phoH, trpA/B/C/D, astB/E, dusC, cysK/D, garK/D, lldP/R/D, yjcB* and *cpxP*) which again depicts that there is a common response between all variants to silver nitrate. If we then compare mutants to one another, in DMB there are 889 genes expressed in common which accounts for ∼50–60% of the genes being expressed and in LB there are 52 genes in common accounting for ∼3–25% of individual total expression. Despite this large increase in the number of genes expressed with the addition of silver nitrate, the most significant overlap in expression occurs within each single mutant with and without silver (50%–80%) indicating that these adaptive mutants confer a response that closely mimics exposure to silver nitrate even in its absence.

### Adaptive mutations in *cusS* are pleiotropic and lead to unique cellular responses

**FIGURE 5:**
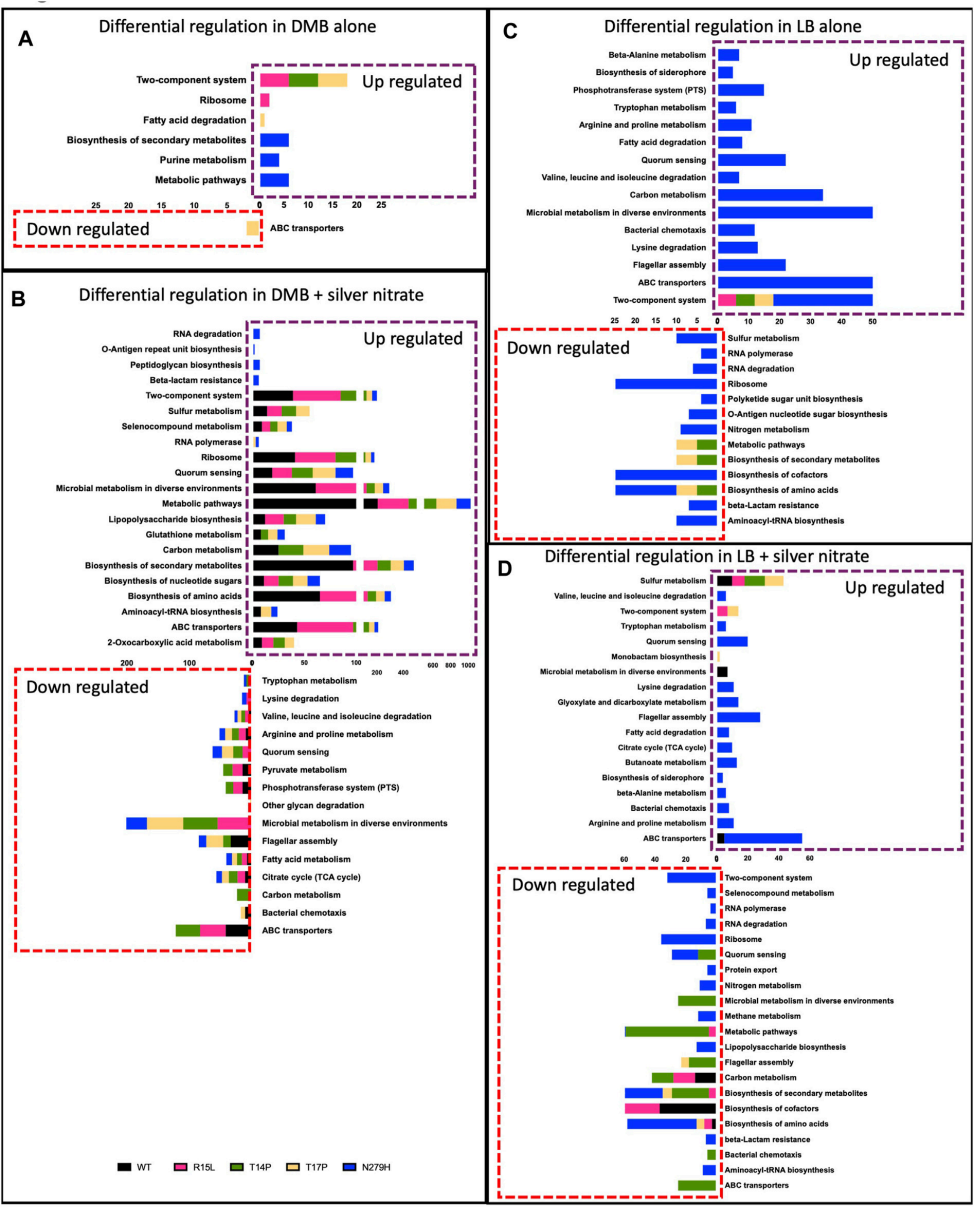
Single mutations in *cusS* are pleiotropic and lead to varying cellular responses. Pathway analysis was performed using limma’s (Ritchie et al., 2015) “kegg” functionality. The genes that were considered Up/Down in this analysis were at FDR <.05. Here we show up/down regulated pathways for each mutant in **(A)** DMB, **(B)** DMB, with silver nitrate, **(C)** LB and **(D)** LB with silver nitrate for the WT and each mutant in each media type supplemented with silver nitrate. X-axis represents the number of genes that have been identified as differentially regulated in that pathway. Data shows that there are overlapping biological processes between all populations, while each mutant shows varying pleiotropic effects through differentially regulating a unique subset of secondary pathways that changes with the genotype, media type and the addition of silver nitrate.

We then wanted to evaluate the differential regulation of specific biological pathways associated with these adaptive mutations to better understand the global cellular responses. Therefore, a Kegg Pathway analysis was performed using limma’s [35] “kegg” functionality and the genes that were considered up/downregulated in this analysis were at FDR <.05. We then removed redundant pathways from the list by keeping the most general pathway name to increase readability of the data. Figure 5 depicts each of the general Kegg pathways that were significantly up/downregulated. In DMB (Figure 5A), Kegg clustering showed upregulation for three mutants (R15L, T14P and T17P) in TCRS. It is important to note that the six genes that were upregulated in all three mutants were all 6 *cus* genes (data not shown). R15L also showed upregulation in ribosomal genes, T17P upregulated fatty acid degradation and downregulated ABC transporters while N27H upregulated genes involved in biosynthesis of secondary metabolites, metabolic pathways and purine metabolism. This data differs from LB alone (Figure 5C), where we see upregulation in TCRS pathways (again in the six *cus* genes) as the only upregulated cluster occurring in all four populations. N279H then upregulates an additional 14 pathways and additional TCRS genes. R15L, as with DMB, does not downregulate any pathways, whereas T14P and T17P downregulate pathways involved in general metabolism, the biosynthesis of amino acids, and the biosynthesis of secondary metabolites. N279H also downregulates amino acid and secondary metabolite biosynthesis while also downregulating an additional 10 pathways (Figure 5C). In the presence of silver nitrate in DMB (Figure 5B), there are 11 pathways that are upregulated and 3 pathways that are downregulated in all 5 populations (WT and all 4 mutants). On the other hand, all four mutants decreased expression of genes for metabolism in diverse environments, quorum sensing and valine, leucine, and isoleucine degradation while each of the mutants also having their own unique expression signatures. Interestingly, there were no differentially regulated pathways in the WT alone, indicating that the mutants were responding with the same response as the WT again, with their own unique signatures. In LB (Figure 5D), WT responded by upregulating genes involved in sulfur metabolism, microbial metabolism in diverse environments and ABC transporters while down regulating the biosynthesis of amino acids, biosynthesis of cofactors and carbon metabolism. These patterns of expression were not as closely mimicked by the mutants which contrasts with what we saw in DMB. R15L upregulated TCRS genes and sulfur metabolism and downregulated the biosynthesis of amino acids, metabolism, and biosynthesis of secondary metabolites. T14P only upregulated sulfur metabolism and downregulated 8 additional pathways, while T17P upregulated sulfur metabolism, TCRS genes and monobactam synthesis while downregulating flagellar assembly genes, the biosynthesis of secondary metabolites and amino acids. Finally, N279H upregulated TCRS and 14 unique pathways. Together, this shows, in absence of silver nitrate, the three N-terminal mutants show very few changes in global cell regulation on top of the common increase in the *cusS* genes. This is opposed to the one C-terminal mutant which shows several changes in differential pathway regulation. These expression trends are conserved as the media is changed to LB, although with there being an overall increase in the total number of global cellular pathways differentially regulated in this rich media. In the original selective environment (DMB +50 ng/mL silver nitrate), the overall response of the adaptive mutants more closely mimics that of the WT with only a few changes. All four mutants reduce expression of genes involved in microbial metabolism in diverse environments, valine, leucine and isoleucine degradation and quorum sensing while each mutant then differentially regulates several unique pathways. As we change the media to LB and add silver nitrate, we see very little conservation between the WT and mutant responses except that R15L, T14P and T17P also increase sulfur metabolism. R15L most closely mimics the WT as compared to the other mutants differentially regulated 4 of the 6 WT pathways. This supports our phenotypic data and demonstrates the importance of the original selection environment to maintain adaptive gene expression patterns.

## Discussion

Evaluating and predicting GxE interactions has relevance to understanding how TCRS contributes to adaptation, and this has the potential to impact clinical treatment interventions. Most clinical studies measuring the fitness of resistant variants do so in just a single laboratory based nutrient environment (Bennett and Lenski, 1997; Schrag SJ et al., 1997; Hall, 2013). Unfortunately, these unidimensional environments are very different from the complex environments that led to selection of the adaptive variants in nature. Thus, clinical tests evaluating resistance phenotypes provide results that are significantly different from those that are true to the adaptive mutants in their original selective environments. Our data supports that gene expression and as a result fitness of our TCRS adaptive mutants is optimal in the original environment in which they were selected. Unfortunately, as we deviate from that environment, fitness changes in an unpredictable manner since rank order, expression levels of target genes, nor global gene expression patterns are maintained.

### Evidence supporting the maintenance and occurrence of R15L in the original selection experiments

Three independent experimental evolution studies have shown that adaptation to silver nitrate and silver nanoparticles select for point mutations in the *cusS* gene (Graves et al., 2015; Randall et al., 2015; Tajkarimi et al., 2017). In one of those studies EE, R15L was found in 11 of the 13-replicates and in 8 of those populations it was found at an *f* = 1.000. Here it also generally shows the highest fitness over all the other mutants. First, in the original selection experiment the main factor leading to successful transfer was 24-hour O.D. values, as daily transfers happened at this time point equivalent. Here, we showed that at 24-h, R15L had a final O.D. that was significantly greater than the WT indicating that it would have likely dominated the serial transfer. We hypothesize that there are several factors contributing to this increased selection. First, R15L shows the greatest expression of *cusS* and *cusR* in DMB supplement with silver nitrate which also results in the greatest expression of the *cusCFBA* operon. Second, we know that the secondary response (prior to the addition of silver nitrate) is already prepped for oxidative stress and downregulation of copper response genes. Third, we would predict that its tertiary response is better refined to improve fitness at this concentration of silver nitrate over the other mutants. When we compared this to the other mutants in the original EE study, N279H, the only C-terminal mutant deviates the most from the N-terminal variants generally showing the lowest fitness in DMB, broth and agar, this may be why it only appeared in one of the 13-replicates and only at a frequency of mutation (*f*) = 0.572 in the original selection experiment. Again, in the original experiment, T14P was also found in a single population and at a low *f* = 0.231 and accompanied by an R15L mutation in the same population at an *f* = 0.769. Changes in fitness shows that in DMB across most concentrations of silver nitrate, these two mutants have similar fitness levels. This similarity in fitness may indicate as to why these two mutations can co-exist within the same population. This also shows that generally, these two mutants, which both reside in the N-terminal tail, show similar results in terms of both fitness and function. T17P which is found in the first transmembrane region, show similarities with R15L and T14P in terms of function, although show variability in fitness. In DMB, T17P has similar fitness to that of N279H although this mutation originates from an experiment that sequenced single clones therefore the frequency of these mutations and the number of replicates from which it came was not identified [13]. Overall, our data supports and provides evidence for why natural selection was imparted on R15L under these experimental conditions.

### The adaptive mechanism resulting from *cusS* mutations is the result of variable GxE interactions between each specific mutation and each specific environment

**FIGURE 6:**
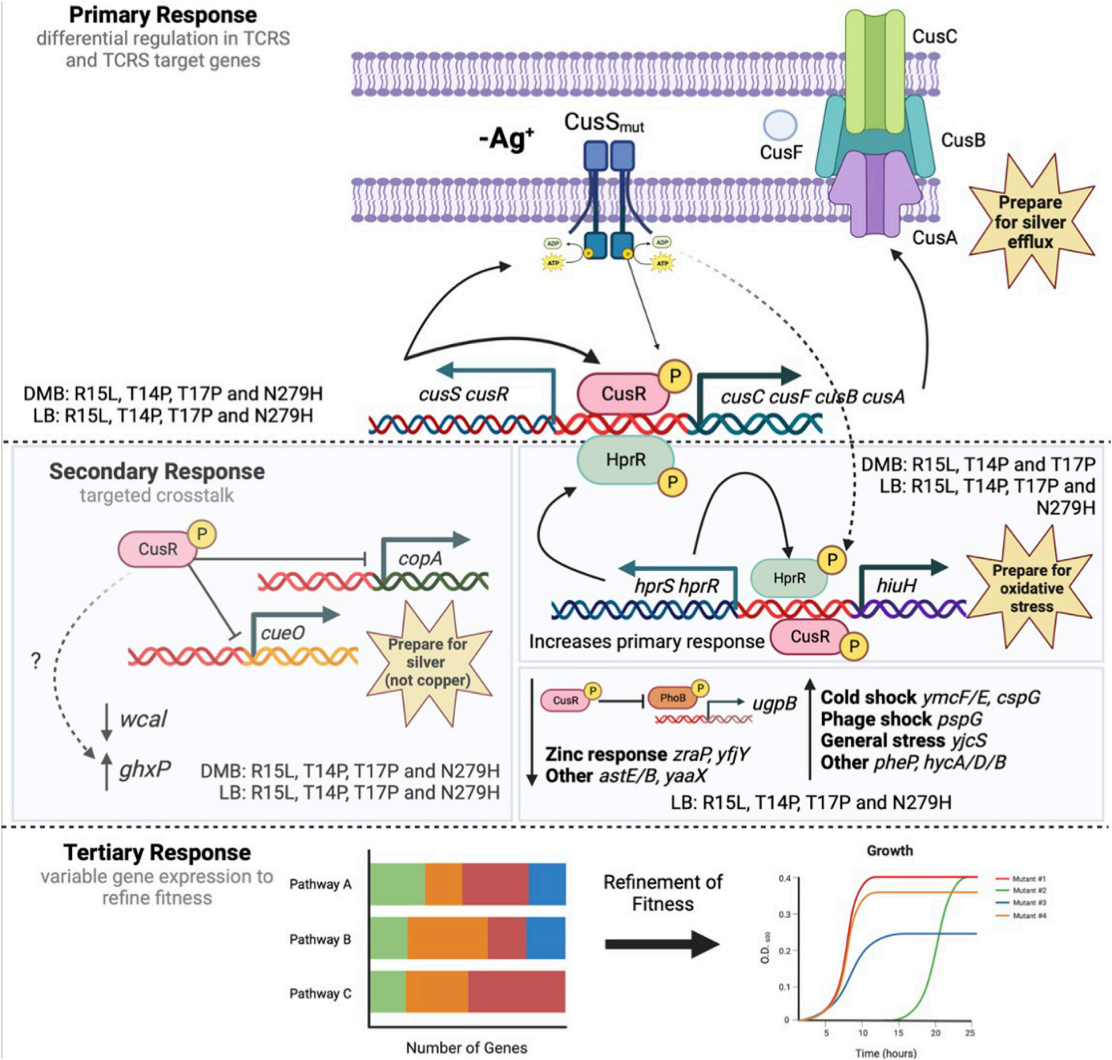
Three-step model depicting the adaptive response of *cusS* silver resistant mutants. Here we propose a 3-step adaptive mechanism associated with adaptation in TCRS genes. This begins with a primary response where mutations in a HK result in increased TCRS expression and constitutive expression in response genes. In this case that is an increased expression of *cusS/R* and constitutive expression of *cusCFBA* although expression levels do vary by mutant due to varying GxE interactions. This primary response then controls a secondary response that leads to crosstalk between the RR and non-cognate pathways. Here we see some pathways that are upregulated by all mutants in both types of media, while other pathways are only upregulated by select mutants in select media. Both the primary and secondary responses will come at a fitness cost therefore we propose that fitness is then refined by unique gene expression patterns associated with the individual GxE interactions deemed the tertiary response. We propose that this mechanism may be maintained by all TCRSs that acquire adaptive mutations provided evidence to support the role that TCRS play in environmental adaptation. Figure was created with BioRender.com.

CusS is a histidine kinase that is part of a TCRS with the response regulator CusR and upon activation, this system induces expression of the CusCFBA silver/copper efflux pump. Upon exposure to silver nitrate, it has been shown in *E. coli*, that these divergently expressed operons are both regulated by CusR, which after activation via phosphorylation, binds a *cusR* box in the intergenic region, turning on transcription of both operons (Franke et al., 2003; Yamamoto and Ishihama, 2005). Based on our analysis of fitness and gene expression, we propose the following model for a TCRS adaptive mechanisms (Figure 6): In absence of silver nitrate (our stimulus) all four *cusS* mutants downregulate *wcal, copA,* and *cueO* while upregulating *ghxP, cusR* and *cusC.* The upregulation of *cusR* and *cusC* is consistent with CusS mutants adopting an active form, allowing for autophosphorylation even in absence of silver. This would then induce activation of CusR via transphosphorylation. Once phosphorylated, CusR can then bind to *cusR* boxes to both auto upregulate and induce expression of the target *cusCFBA* operon. This differential regulation in both the TCRS and target response genes establishes the primary response which varies due to unique GxE interactions for each mutant in each respective environment. The three N-terminal mutants also upregulate *cusS* and *cusR* and this increase in TCRS genes correlates with increased expression in the efflux pump genes. N279H only shows a modest increase in *cusR* expression and as a result only a small increase in efflux pump expression. Thereby showing the importance of *cusR* expression for inducing this primary response which we predict is essential for preemptively preparing the cell for silver efflux. CusR has also been shown to differentially regulate non cognate pathways. In particular, CusR has been shown to downregulate both *copA* and *cueO* when silver is the signal as CusS is more sensitive to silver than it is to copper (Franke et al., 2003; Yamamoto and Ishihama, 2005). Here we see downregulation of both genes in all four of our mutants in DMB. This supports crosstalk between these metal sensing systems even in absence of silver. This conserved crosstalk across mutants establishes the secondary response associated with adaptation in TCRS. We also observed differential expression in both *wcal* and *ghxP* which, to date, have not been linked with CusR activation nor the silver response. WcaL is involved in colanic acid synthesis which is important for slime polysaccharide biosynthesis and GhxP is a high affinity transporter for guanine and hypoxanthine and their role here remains to be elucidated (Papakostas et al., 2013; Scott et al., 2019). The N-terminal mutants also show upregulation in *hprR/S, hiuH* and *pheP*. HprR/S are part of a TCRS that activate *hiuH* expression in response to hydrogen peroxide. It is also known to upregulate itself, *cusS/R* and *cusCFBA* due to sequence and structure similarity between HprR and CusR (Urano et al., 2017). This increased expression may account for the increase in *cusS/R* and *cusCFBA* expression in the N-terminal mutants over N279H. This is supported by the fact that N279H is the only mutant to not over express *hprR/S* and *hiuH* and as a result in DMB, show low efflux pump expression as compared to the N-terminal mutants. Both HprR and CusR have also been shown to collaborate to induce expression of *hiuH* which is also overexpressed in the three N-terminal mutants (Urano et al., 2015; Yamaji et al., 2023). Crosstalk between these systems may be able to help prepare the cell for increased oxidative stress that would be generated by excess metals in the cell via Fenton reactions (Urano et al., 2015). Even though this secondary response generally shows differential regulation in the same genes, each mutant individual response varies thereby again confirming that each mutant displays unique GxE interactions. Finally, each mutant exhibits individualized expression patterns for general cellular pathways that we are deeming the tertiary response. R15L expresses 42 unique genes and is the only mutant to upregulate ribosomal genes. T14P expresses 23 unique genes and shows no individualized changes in global pathway expression. T17P expresses 69 unique genes and upregulates fatty acid degradation and downregulates ABC transporters. N279H uniquely expresses 79 genes and upregulates secondary metabolites biosynthesis, purine metabolism and metabolic pathways. We believe this tertiary response is required to fine tune fitness in an individualized manner through unique GxE interactions to counter the differences between the primary and secondary responses. This is supported by the fact that the mutants do not show a fitness deficit when grown in DMB alone. When silver is then added into the environment, we then begin to see a change in fitness that varies between mutants, in which rank order, again, is not preserved. As we see the greatest overlap in gene expression between each mutant +/- silver nitrate in the same type of media, we know that our mutants are already responding to silver even in its absence. Therefore, we believe that the changes in gene expression between these two environments are mainly to continue to increase the response to silver and cell stress imparted by its presence. At 50 ng/mL, the original selection environment, all mutants show greater fitness via, increased growth rates and final ODs, and we predict that this is due to fact that the mutants have been primed through optimized primary, secondary and tertiary responses for the potential encounter with 50 ng/mL silver nitrate and the variability between mutant response and fitness is the result of the unique GxE interactions.

### Changing the environment alters the GxE interactions

When we switch away from the media in which these mutations were selected, we see maintenance of the primary (expression of the TCRS and efflux pump genes) albeit the extent of the changes is not maintained. As with DMB we see a secondary response in which 23 genes are in common between all four mutants, five of which are in common in absence of silver. This includes *zraP, yaaX, ugpB, yfjY, astE/B, ymcF/E, cspG, hprR, pheP, pspG, hycA/D/B, hiuH, yjcS, cusS/R* and *cusC/F/B/A.* As with *hprR* and *hiuH*, *ugpB* also has a known connection with CusR phosphorylation (Yang et al., 2012), while all the remaining genes have yet to be associated with CusR function, albeit several have connections with metal and stress responses. Finally, we then see fine tuning occurring with the unique tertiary responses associated with each individual mutant. Although this is not optimized as it is with DMB since we see a fitness deficit in all our mutants in LB alone and in presence of silver and we no longer see maintenance of the WT expression patterns as we did in DMB. Together this shows that each mutant phenotype and gene expression patterns are the result of adaptation to that one specific environment and as we change environments each mutant will establish novel GxE interaction that will alter the phenotype and the final response.

Currently, most fitness studies which measure fitness changes in resistant mutants, only vary whether the selective agent is present or not in the nutritional environment (Jin and Gross, 1989; Mariam et al., 2004; Ward et al., 2009). In addition, there are very few studies that have analyzed GxE interactions due to single genomic mutations, thereby removing epistasis from the equation (Yang et al., 2012) and even fewer evaluating this due to mutations within a single gene (Jin and Gross, 1989). Our data shows that adaptation in a particular environment selects for mutants that are specific to the exact environment in which they were selected to optimize GxE interactions. We have been able to show that GxE interactions not only contribute to expression divergence but also to phenotypic plasticity. This has led to the proposal of a 3-step adaptive mechanism associated with adaptation in TCRS genes. This includes a primary response where mutations in a HK result in increased TCRS expression and constitutive expression in response genes. This then controls a secondary response that leads to crosstalk between the RR and non-cognate pathways. Finally, fitness is then refined by unique gene expression patterns associated with the individual GxE interactions. We propose that this mechanism may be maintained by all TCRSs that acquire adaptive mutations provided evidence to support the role that TCRS play in environmental adaptation.

Our data further supports the difficulty associated with creating GxE networks for predicting adaptive phenotypes as our observed phenotypes are not only down to the gene level but vary at the amino acid level. We did observe some consistency in both function and fitness between mutations found in the same domain of a HK (R15L and T14P), albeit currently our data set is too small to make these general conclusions. Moving forward, it will be important to now evaluate this in the context of GxGxE interactions by comparing how fitness is affected due to epistasis in their evolved genetic backgrounds.

## Data Availability

Public access code for the entire Bioproject is accessible through PRJNA1047097 and the individual datasets are also accessible directly through the SRA database using SRX22705802-SRX22705821. https://www.ncbi.nlm.nih.gov/bioproject/?term=prjna1047097.
