# Supplementary material for: Genotype-by-environment interactions govern fitness changes associated with adaptive mutations in two-component response systems
Source: Front Genet. 2024 Feb 23;15:1349507. doi: 10.3389/fgene.2024.1349507 (PMC10920338; doi:10.3389/fgene.2024.1349507)
Supplement: Supplementary file 1 [file DataSheet1.PDF]

24-hour growth curves in DMB

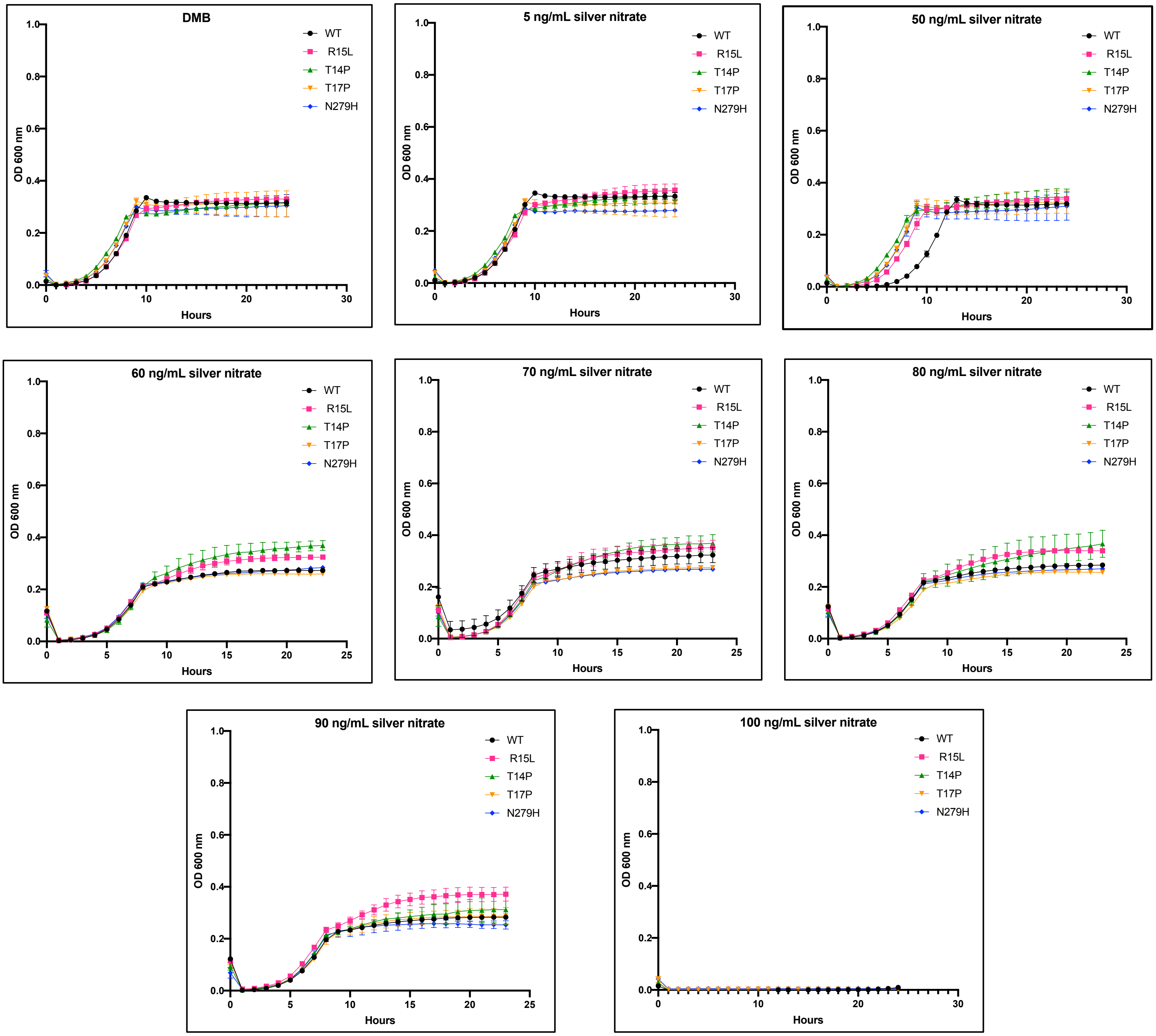

Figure S1: 24-hour growth curves in DMB broth. $O.D._{600}$ was monitored every hour for 24-hours for the WT and each mutant in DMB broth with increasing concentrations of silver nitrate. These curves were then used to determine relative fitness. We calculated the area under the curve (AUC) for the 24-hour growth curves, the AUC along with the standard error and N (N is defined as df + 1 where df is the number of data points for that group minus the number of hours) were then plotted.

Figure S2

24-hour growth curves in LB

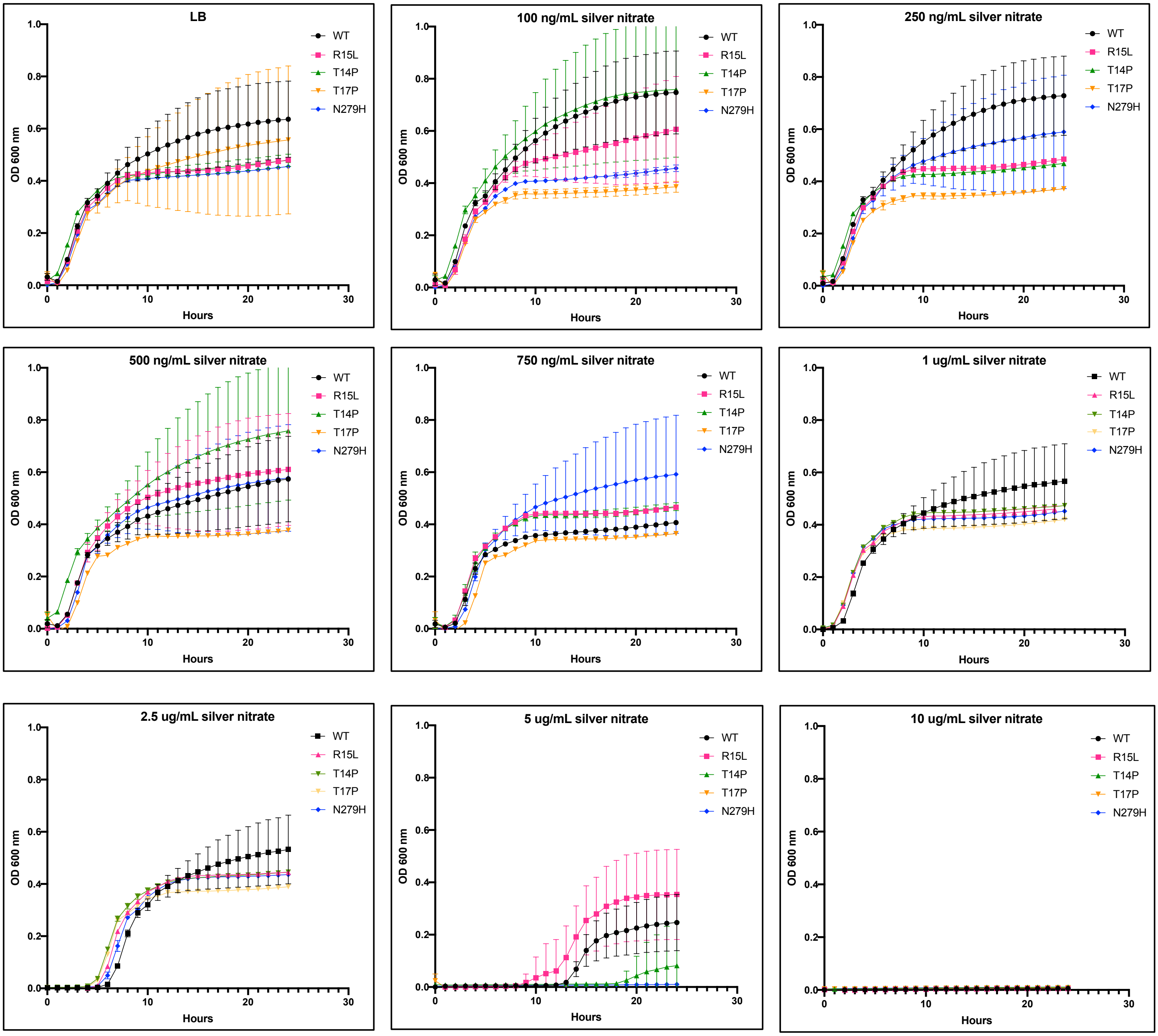

Figure S2: 24-hour growth curves in LB broth. O.D.$_{600}$ was monitored every hour for 24-hours for the WT and each mutant in DMB broth with increasing concentrations of silver nitrate. These curves were then used to determine relative fitness. We calculated the area under the curve (AUC) for the 24-hour growth curves, the AUC along with the standard error and N (N is defined as df + 1 where df is the number of data points for that group minus the number of hours) were then plotted.

Figure S3

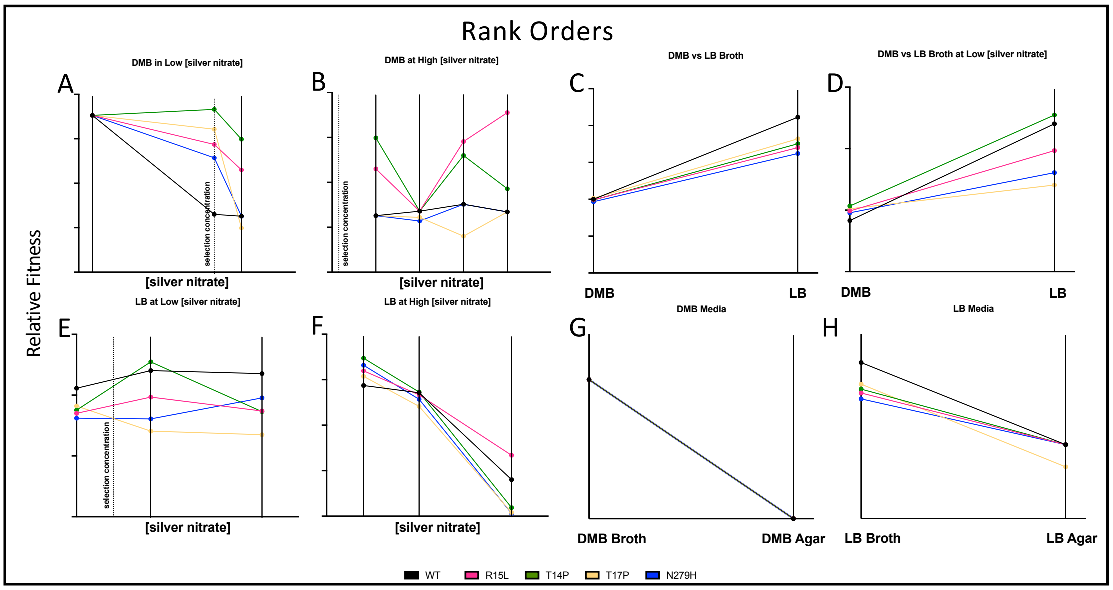

Figure S3: Rank order of the WT and four mutants across tested environments. To better visualize rank order, we used the relative fitness data and for any mutant that was not statistically different from the WT, we changed the relative fitness value to that of the WT. All data that was statistically different from the WT kept its observed values. We plotted the relative fitness from varying environments on the same graph to visualize any changes in rank order.

Figure S4

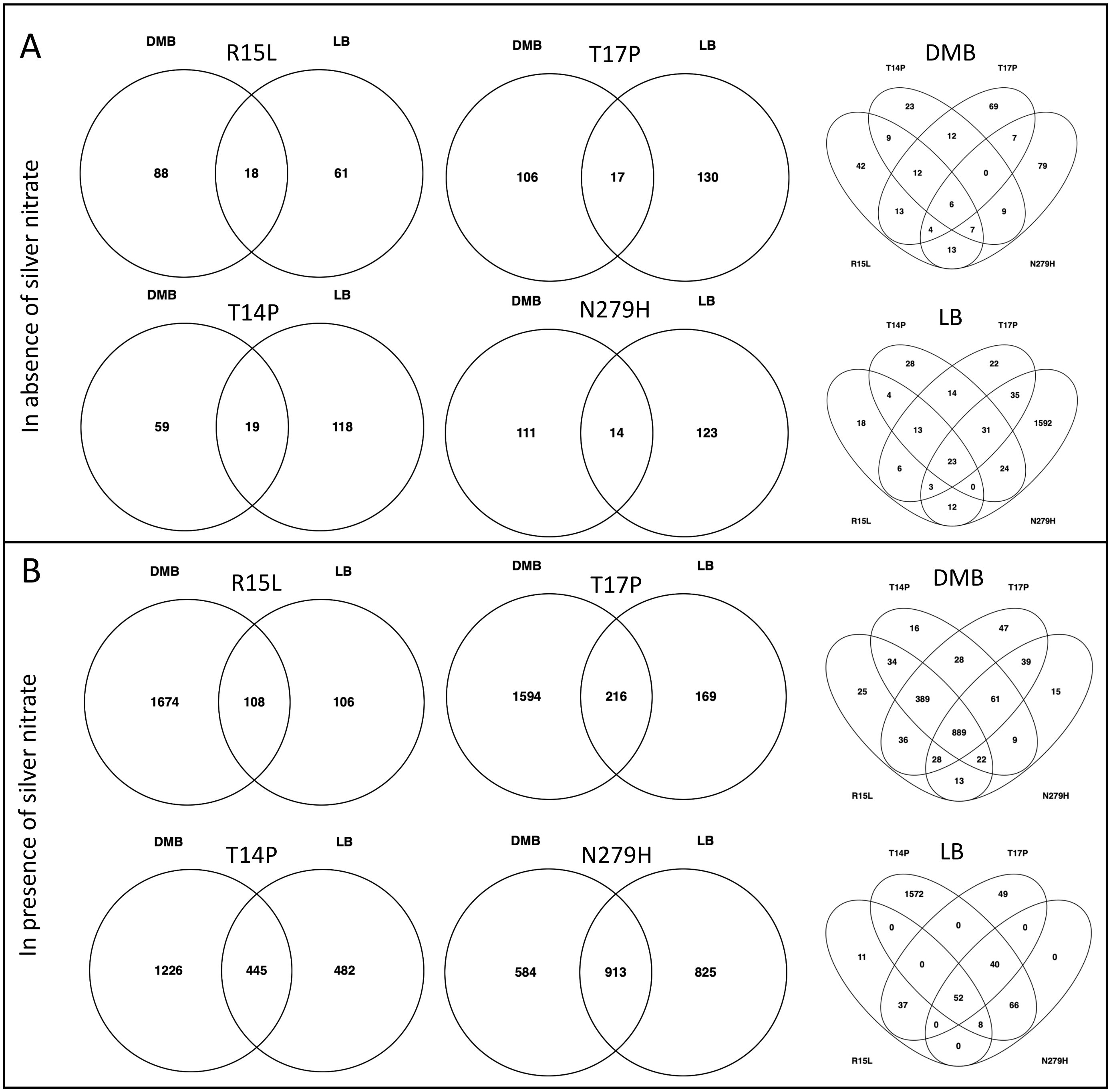

Figure S4. Venn diagrams show overlap between genes expressed in the mutants and the general silver response while also showing unique gene expression patterns for each mutant. Using Venny 2.1 [51] we compared differentially expressed genes in DMB vs. LB between (1) the same mutant and (2) the different mutants. A) Compares differentially expressed genes from each individual mutant, in absence of silver nitrate, in DMB vs. LB. We also compare each individual mutant in DMB to one another and each individual mutant in LB to one another. B) First, gene expression in each mutant, in presence of silver nitrate, was compared to the WT. Common genes were used to create Venn diagrams that compare differentially expressed genes from the individual mutants in DMB vs. LB. Again, we compared each individual mutant in DMB to one another and each individual mutant in LB to one another. These show that these mutations confer substantial changes in global gene expression that varies between mutans and media type. In addition, they can confer a response that mimics exposure to silver nitrate, although not the full response, as additional mechanisms are then activated to fully respond to silver nitrate that are unique to each genotype in each environment.
